# QR-DN1.0: A new distorted and noisy QRs dataset

**DOI:** 10.1016/j.dib.2021.107605

**Published:** 2021-11-20

**Authors:** Milad Monfared, Abbas Koochari, Radin Monshianmotlagh

**Affiliations:** Department of Computer Science, Islamic Azad University Science and Research*,* Tehran, Iran

**Keywords:** Image reconstruction, Image denoising, Geometric distortion, QR

## Abstract

Barcodes are playing a significant role in different industries in the recent years and among the two most popular 2D barcodes, the QR code has grown exponentially. The QR-DN1.0 dataset includes 5 categories of QR codes that will cover low to high density levels. Each group has 15 QR codes: 5 images for testing and 10 images for training. After embedding the QRs into 30 color images using blind watermarking techniques and then extracting the QRs from the images taken with the mobile phone camera with three different methods, we will have three groups of 2250 extracted QR images, which provides a total of 6750 distorted and noisy QR images. In each of the mentioned three categories, the data is divided into two parts: testing, with 750 images, and training, with 2250 images. For every distorted QR in the dataset, a non-distorted instance of it is placed as a ground truth. One of the advantages of this data set is that it is real. Because no simulated noise has been added to the images and this dataset is completely derived from the real word challenge of extracting embedded QRs in color images captured from the watermarked image on the screen. It also includes various types of QRs such as single character, short sentence, long sentence, URL and location.

## Specifications Table

**Table:** 

Subject	Computer Vision, pattern Recognition, Artificial Intelligence
Specific subject area	Image reconstruction, Denoising, Image Watermarking
Type of data	Image
How the data were acquired	All QR images are extracted from 6750 watermarked host color images under the screen-camera process. Watermarked Images are taken from different screens using different mobile cameras, therefore the extracted QR images are distorted due to the screen-camera process.
Data format	Raw
Description of data collection	This dataset consists of 6750 Noisy QRs of 5 group of different QR levels. For each distorted QR image, its original counterpart is placed as the target. There are also three extraction approaches for each data item: simple, quadruple, and voted.
Data source location	• Institution: Islamic Azad University Science and Research, • City/Town/Region: Tehran • Country: Iran
Data accessibility	Repository name: Mendeley Data - QR-DN1.0: A new Distorted and Noisy QRs dataset Data identification number: 10.17632/t2bdr663ms.2 Direct URL to data: https://data.mendeley.com/datasets/t2bdr663ms/2 http://dx.doi.org/10.17632/t2bdr663ms.2

## Value of the Data

• One of the common problems in data related to image reconstruction and noise reduction, especially in the case of QRs, is the lack of data with noise that, like the noise from a screen-camera attack, involves a combination of different noises. Therefore, this is the largest database of distorted and noisy QRs without the addition of simulated noise and This dataset is completely taken from the challenge of extracting and reading QR after the process of capturing a watermarked color image. For each of the three simple, quadruple and voted extraction approaches, 1500 training data and 750 test data are included.
• Using this database, models can be developed based on Supervised learning for QR denoising, QR images reconstruction and QR classification based on the data within them.
• This database can be useful for test and examine noise removal operations, morphing operations and watermarking methods as a complex watermark.

## 1 Data Description

Barcode technology plays an important role in data collection and automated identification technologies. These technologies include coding, symbol representation, printing, identification, data collection and processing. One-dimensional barcodes have been widely used in areas such as the transportation industry, business, manufacturing, home appliances and so on. Today, one-dimensional barcode technology has reached maturity and has played a major role in increasing the speed of data collection and information processing. Traditional one-dimensional barcodes, on the other hand, have limitations such as small capacity for data storage, inability to error correction and dependency on the database. To compensate for these shortcomings, two-dimensional barcodes invited in the early 1990s. A very popular and most common example of this is called QR codes. Extensive applications include storing information such as coordinates and maps, web addresses, print orders, product specifications and prices, and personal details and e-mail addresses [1].

**Fig. 1:**
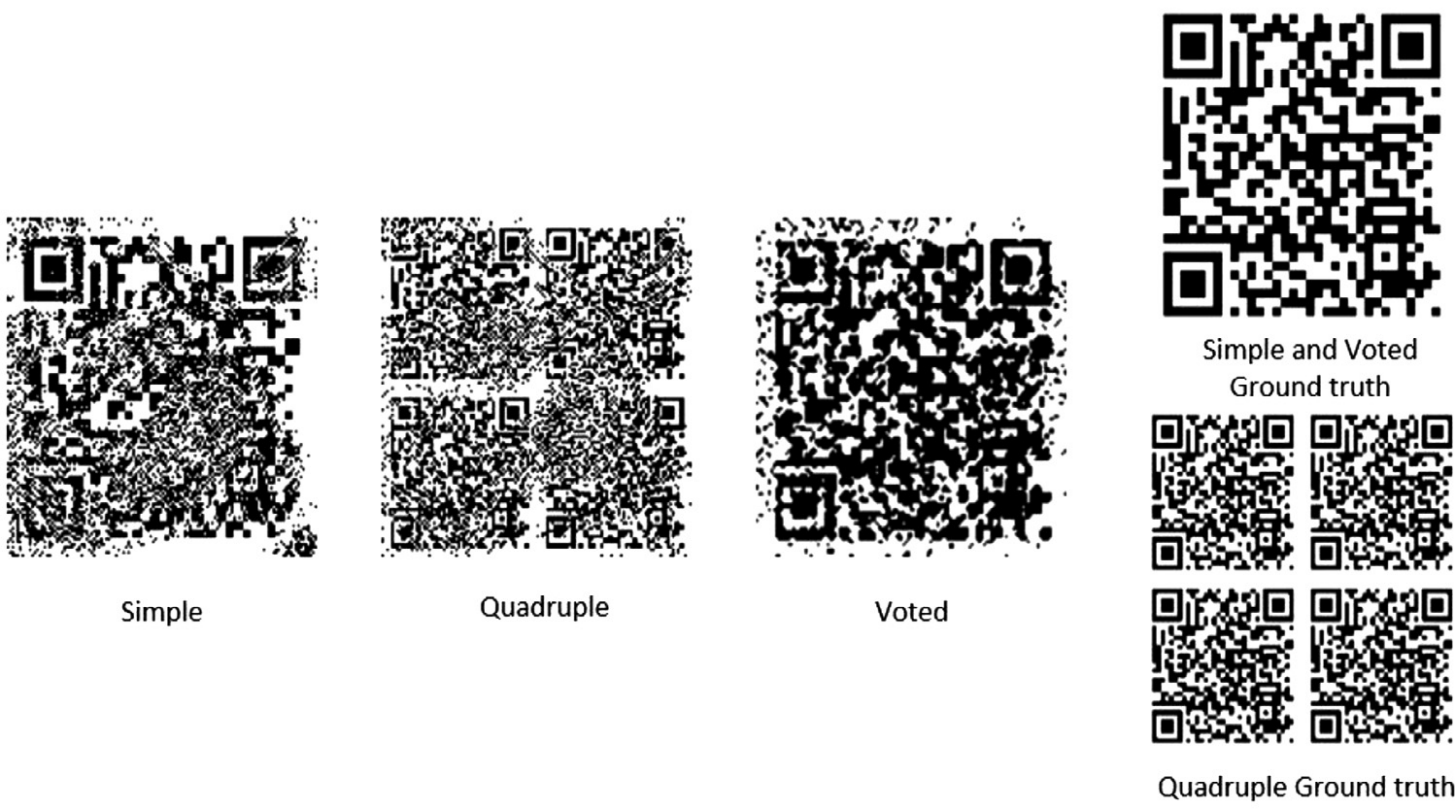
Example of three extraction approaches.

It turns out that these barcodes cover data storage, error correction and database dependency well. Among 2D barcodes, the QR code has a better ability to process encodings in non-English characters and visual information. In addition, the QR code is much faster to read than other 2D barcodes.

In many cases, QR codes may become distorted and noisy. For example, after emending and extracting a QR [2,3] into a color image, the chances of the output being illegible due to geometric distortion and not extracting all QR pixels correctly, are high.

One of the main reasons for collecting this data was a study to extract the QR codes watermarked in color images after the screen-camera attack. During this this research, we encountered the problem of illegibility of QRs extracted from images after the screen-camera attack.

Unlike conventional steganography and watermarking research, in which a logo is usually embedded in the host image and is still recognizable after extraction despite the noise generated, our goal was to extract the QR code in such a way that later from the screen-camera attack, we can extract the data inside it with ordinary QR scanners. But we have seen that the screen-camera attack itself includes various attacks that in addition to creating noise in many cases, also changes the content within the QR and the QR loses its readability.

Many factors are involved in creating this type of distortion, such as image quality, screen quality, camera distance, camera mapping type, lens angle, etc., but our ultimate goal was to read the QRs extracted from the images taken with any camera on any screen to present applied research. To achieve this goal, we used a noise-canceling module based on a deep neural network that required training data to reconstruct distorted and noisy QRs. Noise from a screen-camera attack involves a wide range of noises, and a database was needed to train the neural network in order to teach the character of this type of noise well to the neural network. For this reason, it was not possible to use the artificial noise added to the images to learn the character of the noise caused by the screen-camera attack.

Since the number of QR denoising and reconstruction papers was small and no dataset could be found for real noisy QRs, it was necessary to us to create three independent datasets for all three extraction approaches which are simple, quadruple, and vote-based so that we can have three noise-canceling neural networks for all the approaches in our research. Fig. 1 shows an example of all described approaches.

50 QR codes have been selected for the training phase and 25 QR codes for the test phase, which you can see in the folder (… / QR). Each QR image is embedded in 30 host color images. after watermarking, taking photos [4,5] from watermarked results with different cameras,screens and lighting conditions and then extracting QRs, a dataset with 1500 noisy QR training data and 750 noisy QR test data with resolution of 512 by 512 pixels, was collected. Considering three extraction approaches which are simple, quadruple and voted, we created individual datasets for each approach.2250 training/test samples for each one and 6750 training/test images in total. You can reach each extracting approach in the root folder with separated subfolders for training and test images.

Table 1 shows the five samples of embedded QRs types, the type of data contained in them and the method of naming them in the folder (… / QR).

**Table 1:** Five examples of each of the QR groups used in the dataset.

File name	Kind of data	e.g	data
			
train/lt 1-10 test/lt 1-5	Character		‘1’
			
train/t 1-10 train/t 1-5	Sentence		“this is a QR code”
			
train/u 1-10 rain/u 1-5	URL		https://www.google.com
			
train/l 1-10 train/l 1-5	Location		Milad Tower Location (35°44’41.3”N 51°22’31.1”E)
			
train/ht 1-10 train/ht 1-5	Paragraph		“Steven Paul Jobs (February 24, 1955 – October 5, 2011) was an American business magnate, industrial designer, investor, and media proprietor.”

One of the advantages of QR-DNv1.0 dataset is that it does not simulate noise on images, so that the need to create this data set arose after dealing with the issue of noisy extraction of QR codes from screen captured watermarked images.

All the collected data is the result of extracting embedded QRs from screen captured watermarked color images.

The dataset file contains six sub folders: “extracted one”, “extracted Quad”, “extracted Voted”, “QR”, “target” and “target quad”. the first three are different extracted QRs for simple, quad and voted approaches and each one contains “train” and “test” sub folders. “QR” folder contains original QR files that we watermarked into color images. “target” folder contains ground truth target images for simple and voted approaches and “target quad” contains ground truth target images for quad approach.

**Fig. 2:**
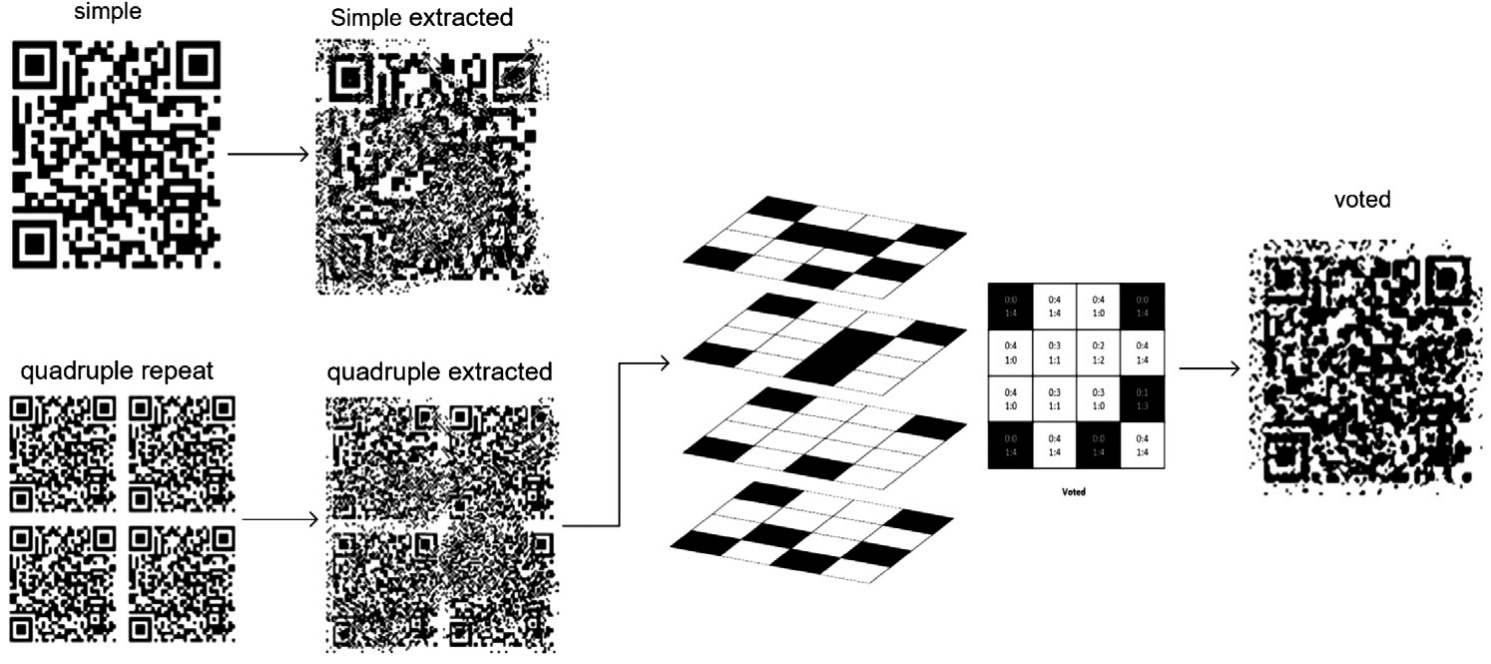
A view of creating three extraction approaches.

**Fig. 3:**
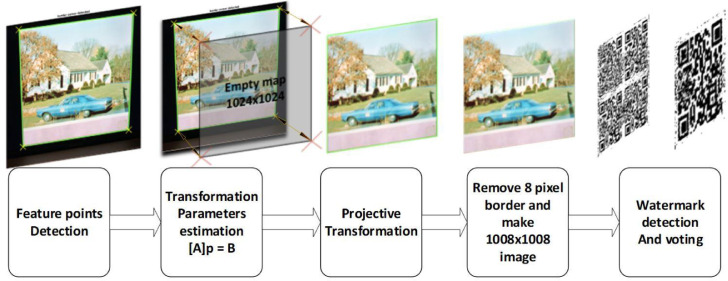
The general schematic of the Extraction process.

This data is available online at Mendeley Repertory. It is structured in a main folder (…/QR images), each subfolder contains train and test data (1500 for train and 250 for test).

Tables 2 and 3 shows the main files of all QR numbered in the train and test folders.

**Table 2:** List of images within the train folder, along with the QR name used.

QR/train/ht-1: 0 to 29	QR/train/l -1: 300 to 329	QR/train/lt -1: 600 to 629	QR/train/t -1: 900 to 929	QR/train/u -1: 1200 to 1229
QR/train/ht-10: 30 to 59	QR/train/l -10: 330 to 359	QR/train/lt -10: 630 to 659	QR/train/t -10: 930 to 959	QR/train/u -10: 1230 to 1259
QR/train/ht-2: 60 to 89	QR/train/l -2: 360 to 389	QR/train/lt -2: 660 to 689	QR/train/t -2: 960 to 989	QR/train/u -2: 1260 to 1289
QR/train/ ht-3: 90 to 119	QR/train/l -3: 390 to 419	QR/train/lt -3: 690 to 719	QR/train/t -3: 990 to 1019	QR/train/u -3: 1290 to 1319
QR/train/ht-4: 120 to 149	QR/train/l -4: 420 to 449	QR/train/lt -4: 720 to 749	QR/train/t -4: 1020 to 1049	QR/train/u -4: 1320 to 1349
QR/train/ht-5: 150 to 179	QR/train/l -5: 450 to 479	QR/train/lt -5: 750 to 779	QR/train/t -5: 1050 to 1079	QR/train/u -5: 1350 to 1379
QR/train/ht-6: 180 to 209	QR/train/l -6: 480 to 509	QR/train/lt -6: 780 to 809	QR/train/t -6: 1080 to 1109	QR/train/u -6: 1380 to 1409
QR/train/ht-7: 210 to 239	QR/train/l -7: 510 to 539	QR/train/lt -7: 810 to 839	QR/train/t -7: 1110 to 1139	QR/train/u -7: 1410 to 1439
QR/train/ht-8: 240 to 269	QR/train/l -9: 540 to 569	QR/train/lt -8: 840 to 869	QR/train/t -8: 1140 to 1169	QR/train/u -8: 1440 to 1469
QR/train/ht-9: 270 to 299	QR/train/l -9: 570 to 599	QR/train/lt -9: 870 to 899	QR/train/t -9: 1170 to 1199	QR/train/u -9: 1470 to 1499

**Table 3:** List of images within the test folder, along with the QR name used.

QR/test/ht-1: 1500 to 1529	QR/test/l -1: 1650 to 1679	QR/test/lt -1: 1800 to 1829	QR/test/t -1: 1950 to 1979	QR/test/ u -1: 2100 to 2129
QR/test/ht-2: 1530 to 1559	QR/test/l -2: 1680 to 1709	QR/test/lt -2: 1830 to 1859	QR/test/t -2: 1980 to 2009	QR/test/u -2: 2130 to 2150
QR/test/ ht-3: 1560 to 1589	QR/test/l -3: 1710 to 1739	QR/test/lt -3: 1860 to 1889	QR/test/t -3: 2010 to 2039	QR/test/u -3: 2160 to 2189
QR/test/ht-4: 1590 to 1619	QR/test/l -4: 1740 to 1769	QR/test/lt -4: 1890 to 1919	QR/test/t -4: 2040 to 2069	QR/test/u -4: 2190 to 2219
QR/test/ht-5: 1620 to 1649	QR/test/l -5: 1770 to 1799	QR/test/lt -5: 1920 to 1949	QR/test/t -5: 2070 to 2099	QR/test/u -5: 2220 to 2249

## 2 Experimental Design, Materials and Methods

As mentioned, in this dataset, 2250 extracted QR samples were collected from all three simple extraction, quadruple and voted extraction approaches.

In the simple watermarking approach, an image of the QR is embedded in the host color image, and after the screen-camera operation, the extracted QR from the image captured with the camera is placed in the data set.

In the quadruple approach, instead of watermarking a QR in the host color image, an image with its quadruple repetition content is used, and after extracting the image taken with the camera, we will have an image of the quadruple QR.

In the voting approach, we use a quadruple repeat image and separate the four QRs from the final extracted image. Vote between the pixels of all four QRs to have a voted QR in the output image. This operation reduces the noise. Fig. 2 provides an overview of how to separate and create three extraction approaches

The watermarking method used is based on the quaternion Fourier transform [3] and changes within its coefficients.

Fig. 3 also shows the steps for extracting QR from a watermarked image.

After taking a picture of the watermarked content from the LCD, the following steps briefly explain how to extract it.

 **Step 1:** Identify the four corners of the image using the margins created around the image.
 **Step 2:** Map the four identified corners of the image on a empty map with a resolution of 1024 by 1024 and calculate each of the 8 coefficients required to reverse the projective transform operation which are located in Eq. (1).
  $$x^{\prime }=\frac{a_{1}x+\;b_{1}y+c_{1}}{a_{0}x+\;b_{0}y+1}$$
  (1) $$y^{\prime }=\frac{a_{2}x+\;b_{2}y+c_{2}}{a_{0}x+\;b_{0}y+1}$$

$(x^{\prime },\;y^{\prime }):\;empty\;map\;1024*1024\;corner\;position$ (x,y):camerapicturecornerposition

 **Step 3:** Perform transform operations and map the pixels using the bilinear technique.
 **Step 4:** Remove the 8-pixel border around the image.
 **Step 5:** Take a photo of the watermark operation (using inverse of quaternion Fourier transform and identify the changed coefficients) and extract the watermarked QR and do the voting if necessary.

## Ethics Statements

Hereby, I Milad Monfared consciously assure that for the manuscript QR-DN1.0: A new Distorted and Noisy QRs dataset the following is fulfilled: 1) This material is the authors' own original work, which has not been previously published elsewhere. 2) The paper is not currently being considered for publication elsewhere. 3) The paper reflects the authors' own research and analysis in a truthful and complete manner. 4) The paper properly credits the meaningful contributions of co-authors and co-researchers. 5) The results are appropriately placed in the context of prior and existing research. 6) All sources used are properly disclosed (correct citation). Literally copying of text must be indicated as such by using quotation marks and giving proper reference. 7) All authors have been personally and actively involved in substantial work leading to the paper, and will take public responsibility for its content. The violation of the Ethical Statement rules may result in severe consequences. To verify originality, your article may be checked by the originality detection software iThenticate. See also  http://www.elsevier.com/editors/plagdetect. I agree with the above statements and declare that this submission follows the policies of Solid State Ionics as outlined in the Guide for Authors and in the Ethical Statement. Date: 20, November 2021

## CRediT Author Statement

**Milad Monfared:** Conceptualization, Methodology, Software, Validation, Formal analysis, Writing – original draft, Visualization; **Abbas Koochari:** Conceptualization, Writing – review & editing, Supervision; **Radin Monshianmotlagh:** Writing – review & editing.

## Declaration of Competing Interest

The authors declare that they have no known competing financial interests or personal relationships that could have appeared to influence the work reported in this paper.
